# Crab spiders impact floral-signal evolution indirectly through removal of florivores

**DOI:** 10.1038/s41467-018-03792-x

**Published:** 2018-04-10

**Authors:** Anina C. Knauer, Moe Bakhtiari, Florian P. Schiestl

**Affiliations:** 1 Department of Systematic and Evolutionary Botany, University of Zurich, Zollikerstrasse 107, 8008 Zurich, Switzerland; 2 Present Address: Institute of Biology, University of Neuchatel, Rue Emile-Argand 11, 2000 Neuchatel, Switzerland

## Abstract

The puzzling diversity of flowers is primarily shaped by selection and evolutionary change caused by the plant’s interaction with animals. The contribution of individual animal species to net selection, however, may vary depending on the network of interacting organisms. Here we document that in the buckler mustard, *Biscutella laevigata*, the crab spider *Thomisus onustus* reduces bee visits to flowers but also benefits plants by feeding on florivores. Uninfested plants experience a trade-off between pollinator and spider attraction as both bees and crab spiders are attracted by the floral volatile β-ocimene. This trade-off is reduced by the induced emission of β-ocimene after florivore infestation, which is stronger in plant populations where crab spiders are present than where they are absent, suggesting that plants are locally adapted to the presence of crab spiders. Our study demonstrates the context-dependence of selection and shows how crab spiders impact on floral evolution.

## Abstract (web summary)

Crab spiders can reduce pollination and thus have been suggested to select against the floral traits that attract them. Here, the authors instead find evidence of local adaptation of buckler mustard plants to increase attraction of crab spiders by emission of the floral volatile β-ocimene when infested by florivores.

## Introduction

Plant–animal interactions are a major driver of plant evolution, including both local adaptation and species divergence^1–4^. Most plants interact directly with various mutualists (e.g., pollinators) and antagonists (e.g., herbivores and florivores)^3,5–7^, but may also interact indirectly with animals from the third trophic level (e.g., predators and parasitoids)^8^. The fitness outcome of such indirect plant–animal interactions depends on whether the involved animals kill mostly plant mutualists or antagonists. Plants can communicate simultaneously with all of these animals via floral signals^9^. Considering pairwise interactions, signals that attract mutualists but deter antagonists should be favored by natural selection. Net selection on plant traits, however, is shaped by the entire network of interacting organisms^10–13^, as the fitness effect of one interaction can depend on the presence or intensity of another interaction^3,6,14^. For example, many floral signals have evolved to attract mutualists, mainly pollinators^15^, but antagonists can eavesdrop on such signals and use them to their own advantage causing a trade-off between the attraction of mutualists and the avoidance of antagonists^16–18^. This is the case in the wild Texas gourd which attracts a specialist pollinator and a specialized herbivore by the same floral volatile^16^. Further, the same predator may affect plant fitness differently when feeding on plant mutualists than when feeding on plant antagonists, which can depend on the relative abundance of these mutualists and antagonists.

**Fig. 1:**
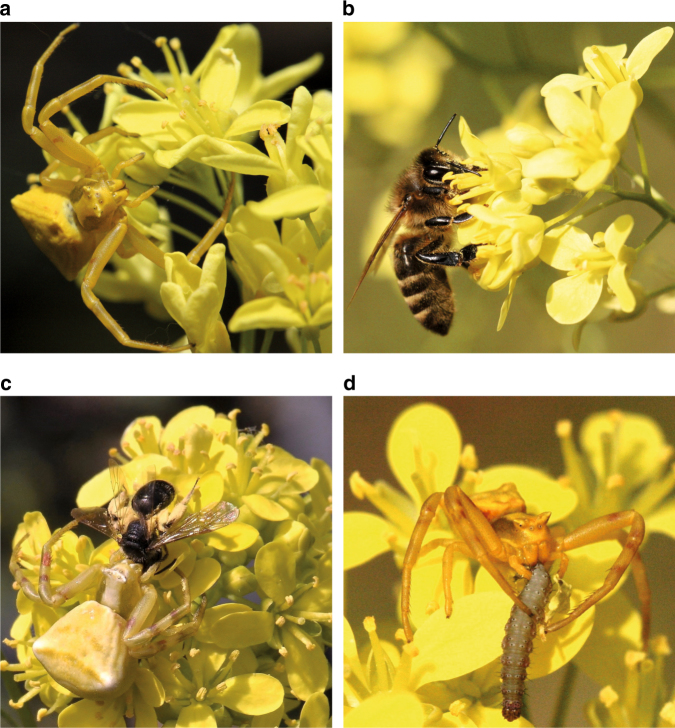
The buckler mustard (*Biscutella laevigata*) and various interacting organisms. **a** A crab spider (*Thomisus onustus*) on *B. laevigata* flowers. **b** A honey bee (*Apis mellifera*) visiting *B. laevigata* flowers. **c** A crab spider feeding on a wild bee (*Halictus* sp.) **d** A crab spider feeding on a florivore *(Plutella xylostella)*. All photos by A.C. Knauer

Crab spiders camouflage themselves on flowers to hunt flower-visiting insects such as pollinators (Fig. 1)^19^. Despite the spiders’ camouflage, pollinators, especially bees, can visually detect crab spiders and avoid spider-occupied flowers. Therefore, crab spiders are thought to have a negative effect on plant fitness^20–22^. Both crab spiders and bees are attracted to flowers by scent^23^, suggesting an eavesdropping scenario in which crab spiders exploit pollinator-attracting signals to find prey. However, because floral scent comprises complex blends of volatile compounds, it is still unknown whether spiders use the same volatiles as pollinators to find flowers and whether plants experience a trade-off between the attraction of bees and the avoidance of crab spiders. Furthermore, crab spiders are generalist predators and sometimes also feed on insect herbivores (e.g., Fig. 1d), a phenomenon that can reduce flower damage and increase plant fitness^24,25^. The net effect of generalist flower-dwelling predators on plant fitness can be positive or negative, according to mathematical models, depending on the relative abundance of mutualists and antagonists in a plant population^26^. However, the net effects of crab spiders on plant fitness and their impact on floral trait evolution remains little studied^24,25,27^.

In this study, we investigate the effect of crab spiders on plant interactions with pollinators and florivores and the consequences for floral trait evolution in the alpine herb *Biscutella laevigata* (Fig. 1). In the Swiss and Italian regions where this study was conducted, the crab spider *Thomisus onustus* occurs in lowland populations on up to 30% of plants, but is absent from highland populations. Lowland and highland plant populations within each region share an evolutionary origin and belong to the same genetic lineage^28^. In the spider-associated populations, plants are mainly pollinated by bees and florivore-infestation can reach up to 47% of plants. We address the following specific questions: (1) Does *B. laevigata* experience a trade-off between the attraction of pollinators and the avoidance of crab spiders? (2) How does the presence of crab spiders (*Thomisus onustus*) affect pollinator attraction by floral traits? (3) What is the fitness effect of *T. onustus* on *B. laevigata* in the absence or presence of florivores? (4) Does *B. laevigata* induce a crab spider-attracting scent compound when infested with florivores? (5) Are *B. laevigata* populations locally adapted in their indirect defense to the presence of the crab spiders? We found that crab spiders on inflorescences reduced bee visitation to flowers. However, crab spiders did benefit *B. laevigata* plants when infested by florivores by eating and thus removing them from the flowers. Plants were also more attractive to crab spiders after florivore infestation because florivory induced β-ocimene, implying this plasticity is an adaptation of *B. laevigata* plants to attract crab spiders.

## Results

### Interaction between crab spider and pollinator attraction

The effect of crab spiders on plant-pollinator interactions was studied in the Swiss lowland population. In this population, 26.3 ± 0.9% (mean ± s.e.) of plants had a crab spider on their inflorescence. Bees (Apidae) represented the main pollinators with respect to visitation frequency and pollen carryover (Supplementary Tables 1, 2). We found no evidence for pollen limitation, as supplemental hand-pollination did not increased the fitness of plants (two-sample *t*-test, *t* = 1.519, *P* = 0.16).

**Fig. 2:**
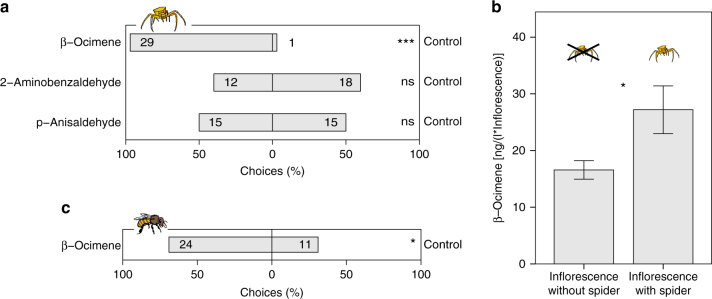
Preferences for floral scent compounds in crab spiders and bees. **a** Dual-choice behavioral assays testing the preference of crab spiders for the three main floral volatiles emitted by *Biscutella laevigata* against an odorless control. Numbers in bars are the absolute numbers of crab spiders selecting either side. Binomial test: ns: *P* > 0.05, *P < 0.05, **P < 0.01, ***P < 0.001. **b** Emission of β-ocimene in plants with and without crab spiders hunting on inflorescence of *B. laevigata* in the Swiss lowland population (*N* = 94). Each bar represents a mean ± s.e., significant differences between treatments are indicated by an asterisk. **c** Dual-choice behavioral assays testing the preference of bees for *B. laevigata* plants with augmented emission of β-ocimene against a control plant. Numbers in bars are the absolute number of landings on inflorescences. Binomial test: ns: *P* > 0.05, *P < 0.05, **P < 0.01, ***P < 0.001

In dual-choice behavioral assays testing the attractiveness of the three main floral scent compounds, crab spiders showed a significant preference for the floral monoterpene β-ocimene over an odorless control (binomial test, *P* < 0.001) (Fig. 2a). In contrast, we detected no preference for two aromatic compounds, p-anisaldehyde (binomial test, *P* = 1) and 2-aminobenzaldehyde (binomial test, *P* = 0.36) (Fig. 2a). Consistent with a preference for β-ocimene, crab spider presence on *B. laevigata* inflorescences in the Swiss lowland population was positively associated with the emission of β-ocimene (binomial logistic regression, estimate ± s.e. = 0.04 ± 0.01, *z* = 2.635, *P* = 0.008) (Fig. 2b). On average, plants visited by crab spiders emitted 60% more β-ocimene than plants on which crab spiders were not observed. β-Ocimene also attracted bees: in dual-choice behavioral assays bees showed a significant preference for β-ocimene augmented plants compared to control plants (binomial test, *P* = 0.041) (Fig. 2c).

We used plot experiments to compare bee attraction by floral traits in the presence and absence of crab spiders, which was measured by generalized mixed models with a Poisson distribution. Bees were attracted by high number of flowers (estimate ± s.e. = 0.19 0.03, *z* = 6.55, *P* < 0.001) and large amounts of aromatic scent compounds (estimate ± s.e. = 0.11 ± 0.03, *z* = 3.76, *P* < 0.001) independent of the treatment (Table 1). However, the presence of crab spiders on 25–33% of the plants significantly affected bee attraction by β-ocimene (trait × treatment effect) (Table 1). In the absence of crab spiders, β-ocimene significantly attracted bees (estimate ± s.e. = 0.12 ± 0.04, *z* = 2.87, *P* = 0.004), but when crab spiders were present, no association between bee visitation and β-ocimene could be detected (estimate ± s.e. = −0.06 ± 0.04, *z* = −1.59, *P* = 0.11). Additionally, the time an inflorescence was occupied by a hunting crab spider had a significant negative effect on the number of bee visits (estimate ± s.e. = −0.03 ± 0.01, *z* = −3.015, *P* = 0.003).

**Table 1:** Effect of floral traits and of the interactions between floral traits and crab spider presence on the attractiveness of flowers to bees (crab spiders hunting on 25–33% of the plants or no spiders present, generalized mixed model, *N* = 108 per treatment)

Trait	*χ* ^2^	d	*P* value
β-Ocimene	0.643	1	0.42
Aromatics	14.727	1	**<0.001**
Corolla size	0.005	1	0.94
Number of flowers	42.965	1	**<0.001**
Spiders presence × β-ocimene	10.282	1	**0.001**
Spiders presence × aromatics	2.007	1	0.16
Spiders presence × corolla size	0.630	1	0.43
Spiders presence × number of flowers	0.846	1	0.36

Numbers in bold refer to significant *P* values

### Tritrophic interaction

**Fig. 3:**
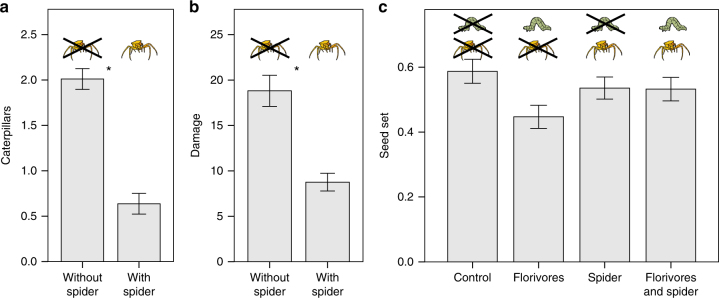
Effect of crab spiders on damage inflicted by florivory. **a** Number of remaining florivores on plants with and without crab spiders on the inflorescence (*N* = 50). Florivores were counted at the end of the day after three caterpillars have been placed on plants in the morning. Each bar represents a mean ± s.e., significant differences between treatments are indicated by an asterisk. **b** Florivore damage in plants with and without crab spiders (*N* = 50). Damage was measured after *Plutella xylostella* feeding for 4 days; it was calculated as the sum of flowers and buds with feeding damage by florivores. Each bar represents a mean ± s.e., significant differences between treatments are indicated by an asterisk. **c** Fitness (measured as seed set) of *Biscutella laevigata* plants under four treatments (*N* = 100): control: no crab spider or florivores; florivores: infestation with florivores; spider: crab spider on the inflorescence; florivores and spider: crab spider on the inflorescence and infestation with florivores. Crab spiders and florivores had a significant interactive effect on plant fitness; crab spiders reduced the negative effect of florivores (mixed effect model: estimate ± s.e. = 0.14 ± 0.06, *t* = 2.205, *P* = 0.027)

We manipulated plant infestation and crab spider presence in a plot experiment to measure the fitness effects of crab spiders and florivores separately and in combination. Additionally we monitored the catches of crab spiders. We found that 89.9% of the recorded captures by the crab spiders were florivores in infested inflorescences (e.g., Fig. 1d). Accordingly, the number of florivores was significantly reduced on plants with a crab spider on the inflorescence compared to plants without a spider (linear mixed model, estimate ± s.e. = −1.4 ± 0.1, *t* = −10.11, *P* < 0.001) (Fig. 3a). Total floral damage was also significantly lower for plants with spiders than for plants without spiders (linear mixed model, estimate ± s.e. = −10.1 ± 1.7, *t* = −5.871, *P* < 0.001) (Fig. 3b). While the presence of crab spiders on inflorescences did not affect the plant’s female fitness (seed set) (linear mixed model, estimate ± s.e. = −14 ± 8, *t* = −1.855, *P* = 0.63), florivores had a significant negative fitness effect (linear mixed model, estimate ± s.e. = −24 ± 8, *t* = −3.138, *P* = 0.021). Moreover, we found a significant interaction between spider and florivore presence on plant fitness; crab spiders reduced the negative effect of florivores on relative fitness (linear mixed model, estimate ± s.e. = 23 ± 11, *t* = 2.124, *P* = 0.034) (Fig. 3c). In the Swiss lowland population of *B. laevigata*, 2 out of 17 recorded prey items were florivores (12%).

**Fig. 4:**
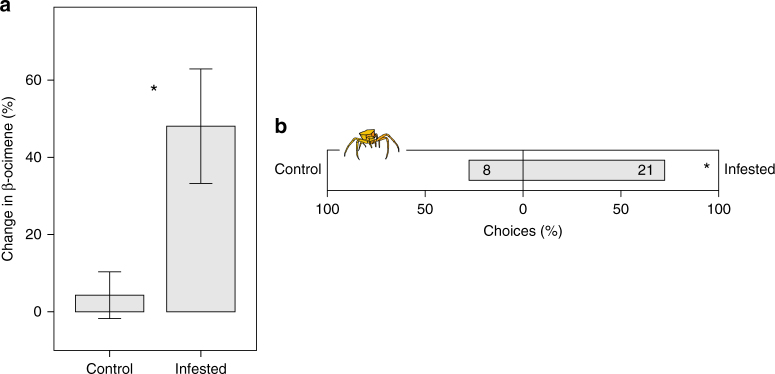
Tritrophic interaction between *Biscutella laevigata*, florivores and crab spiders. **a** Inducibility of β-ocimene in florivore-infested and control plants (*N* = 68). Each bar represents a mean ± s.e., significant differences between treatments are indicated by an asterisk. **b** Dual-choice behavioral assays testing the preference of crab spiders for florivore infested *B. laevigata* plants against a control plants. Numbers in bars are the absolute number of choices of inflorescences. Binomial test: ns: *P* > 0.05, *P < 0.05, **P < 0.01, ***P < 0.001

After florivore infestation, the emission of β-ocimene, the main spider attractant, significantly increased by 44 ± 16% compared to control plants (linear mixed model, estimate ± s.e. = 1.4 ± 0.4, *t* = 3.555, *P* < 0.001) (Fig. 4a, see also Supplementary Table 3 for inducibility of other compounds). Correspondingly, in dual-choice behavioral assays, crab spiders showed a significant preference for infested plants over control plants (binomial test, *P* = 0.024) (Fig. 4b). Also, in the natural plant population, crab spiders occurred significantly more often on infested plants than expected by chance (binomial logistic regression, estimate ± s.e. = 0.008 ± 0.003, *t* = 2.858, *P* = 0.005). 69% of the plants selected for hunting by crab spiders were infested with florivores, although the infestation rate of the whole population was only 47%. Florivory significantly reduced plant fitness by 45% on average (*t*-test, *t* = 2.2357, *P* = 0.031), which was measured in the Swiss highland population where spiders are absent (infestation rate reached 36%).

### Local adaptation to crab spiders

**Fig. 5:**
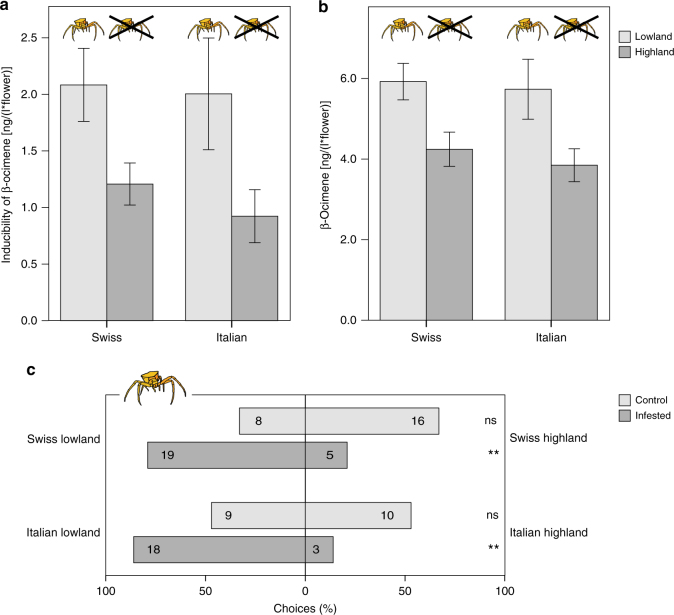
Adaptation of *Biscutella laevigata* populations to crab spiders. **a** Inducibility of β-ocimene emission in lowland (with spiders) and highland (without spiders) populations, in the Swiss and Italian lineage (*N* = 110). Inducibility was significantly higher in lowland populations than in highland populations (linear model: estimate ± 0.09 ± 0.04, *t* = 2.034, *P* = 0.044). The lineage (Swiss vs. Italian) on the other hand did not affect the inducibility of this compound (*t* = 0.565, *P* = 0.57). **b** Absolute emission of β-ocimene after florivore infestation in low- and highland populations, in the Swiss and Italian lineage (*N* = 110). Lowland populations emitted significantly higher amounts of β-ocimene compared to highland populations (estimate ± s.e. = 1.7 ± 0.7, *t* = 2.444, *P* = 0.016). The lineage (Swiss vs. Italian) on the other hand had no effect on the amount of β-ocimene (*t* = 0.545, *P* = 0.59). **c** Dual-choice behavioral assays testing the preference of crab spiders for lowland *B. laevigata* plants against highland plants, for the Swiss and Italian lineage separately. Numbers in bars are the absolute number of crab spiders selecting each side. Binomial test: ns: *P* > 0.05, *P < 0.05, **P < 0.01, ***P < 0.001

We found a significant effect of altitude (lowland vs. highland) on the inducibility of β-ocimene emission (linear model, *t* = 2.034, *P* = 0.044) (Fig. 5a). Inducibility was approximately twice as high in lowland populations as in highland populations. In contrast, region (Swiss vs. Italian) did not affect the inducibility of β-ocimene (linear model, *t* = 0.623, *P* = 0.53) (see also Supplementary Table 3 for inducibility of other compounds). Further, prior to infestation with florivores, the absolute constitutive emission of β-ocimene did not differ between altitudes or regions (linear model, altitude: *t* = 1.682, *P* = 0.10; region: *t* = 0.132, *P* = 0.90). After infestation, however, lowland populations emitted significantly higher amounts than did highland populations (linear model, estimate ± s.e. = 1.7 ± 0.7, *t* = 2.444, *P* = 0.016), while region still did not affect β-ocimene emission (linear model, *t* = 0.545, *P* = 0.59) (Fig. 5b).

In dual choice behavioral assays, crab spiders showed no preference for lowland over highland plants in the absence of florivores in either the Swiss (binomial test, *P* = 0.15) or the Italian lineage (binomial test, *P* = 1). After florivore infestation, however, crab spiders significantly preferred lowland over highland plants in both regions (binomial test, *P* = 0.007 for Swiss lineage and *P* = 0.001 for Italian lineage) (Fig. 5c).

## Discussion

Complex webs of plant–animal interactions can give rise to conflicting and nonadditive selection pressures, with consequences for the evolution of floral signals. The effect of indirect plant–animal interactions on flower evolution is little known and little explored for plants and crab spiders, despite their common occurrence on flowers. Here we show nonadditive effects of crab spiders and florivores on plant fitness, with spiders having a net positive effect on plant fitness through their removal of florivores. We also show that plants are locally adapted to attract crab spiders through induced floral volatile emission following herbivore attack. Our results demonstrate the relevant effect of crab spiders in driving floral trait evolution in *B. laevigata* and give insights into patterns of selection imposed on floral traits by individual components and whole plant–animal interactions webs.

As many crab spiders hunt specifically on flowers, their effects on plant fitness has so far mostly been considered in connection to reducing pollinator visits^20,29,30^. Signals attracting pollinators are especially relevant for crab spiders as pollinators form a considerable proportion of their diet. Identifying β-ocimene as an attractive signal for both pollinators and crab spiders suggests that crab spider preference has evolved to exploit the established communication channel between plants and pollinators, facing plants with a trade-off between the attraction of pollinators and the avoidance of antagonists. Similar to our results, in *Chrysanthemum frutescens* flowers, the crab spider *Thomisus spectabilis* and honey bees overlap in their preferences for floral symmetry as well as floral scent^23,31^. Such exploitative preferences should be under strong selection to maximize prey encounter in “sit-and-wait” predators as this strategy is only effective when sufficient numbers of prey pass within striking distance of predators. A preference for β-ocimene might be especially successful as this compound is attractive to various pollinator taxa and is one of the most common floral volatiles, emitted from 71% of plant families^32–36^. Many bee species, however, can visually identify crab spiders on flowers, which decreases flower approaches^20,29^. Accordingly, we found reduced visitation to inflorescences with crab spiders, which disrupted the association between pollinator visitation and the amount of the floral volatile β-ocimene. Although seed production is barely pollen-limited in *B. laevigata*, pollinators may still mediate selection via the male component of fitness^37–39^. The presence of crab spiders may thus change pollinator-mediated selection on floral traits.

Various plant traits have evolved to reduce the negative effect of herbivory on plant fitness, including indirect defense^8,40–43^. In *B. laevigata*, crab spiders are potentially detrimental for plant fitness in the absence of florivores, but they become beneficial when florivores are present. This pattern suggests that plants are under selection to attract spiders only when attacked by florivores, which we confirmed by showing higher attractiveness of infested plant for spiders, likely mediated by induced floral β-ocimene emission. Indeed, β-ocimene is involved in many tritophic interactions as an attractant of natural enemies^44–47^. However, we cannot preclude the possibility that the inducibility of β-ocimene has evolved as a direct defense against herbivores, as this compound has been documented to reduce oviposition in two herbivore species^48,49^. In *B. laevigata*, both interactions (with florivores and with crab spiders) may have selected for the inducibility of β-ocimene. Furthermore, β-ocimene is one of the most common herbivore-induced plant volatiles and can be induced not only in flowers but also in leaves. After leaf damage by herbivory, the biosynthesis in leaves is “de novo”^33,44,50–53^, which may enable crab spiders to find leaf herbivores. Thus, the beneficial effect of the crab spider *T. onustus* on plant fitness might also occur in several other plant species that spiders use for hunting.

Several studies have demonstrated local adaptation in plants to the presence or abundance of herbivores through induction of direct defense^54–58^. In contrast, local adaptation of plant populations to indirect defense across trophic levels is largely enigmatic. Our study provides evidence for local adaptation of *B. laevigata* populations to the tritrophic interaction between plant, florivores and the crab spider *T. onustus* through induced β-ocimene emission. The model by Higginson et al.^26^ predicted the evolution of predator-attracting traits under conditions that positively influence the net effect of crab spiders on plant fitness: high pollinator abundance and effectiveness, and strong florivory. These conditions are met in our *B. laevigata* populations, where florivore infestation reached up to 47% and decreased fitness by 45% in infested plants. Also, buckler mustard populations with crab spiders are unlikely to be pollinator-limited, as pollinators are usually abundant and the low number of ovules per flower (*Biscutella* species have fruits with two seeds)^59^ makes a few pollen grains sufficient for full seed set (see Supplementary Table 2 for pollinator effectiveness). Finally, the proportion of florivores in the prey pool of crab spiders was 12% in the Swiss lowland population, indicating an occasional positive effect by predators on plant fitness.

In conclusion, our data suggest a so far overlooked role of crab spiders in floral trait evolution. Compared to other natural enemies of herbivores, crab spiders should impose strong selection on plants in a tritrophic system, as they can remove several florivores per day and—unlike parasitoids—kill their prey immediately. In addition, crab spiders occur worldwide with over 2000 species and are commonly found on flowers^60–62^. Thus, their impact on plant evolution may be widespread among angiosperms.

## Methods

### Study system

*Biscutella laevigata* ssp *laevigata* (Brassicaceae) is a perennial herb native to the central parts of the Alps, where it occurs from about 500 to over 2000 m above sea level (m.a.s.l.). We conducted our study in four populations of *B. laevigata*: two in Switzerland and two in Italy. Although they belong to the same tetraploid subspecies, populations in the Swiss region (lower Rhone valley) belong to a different lineage than the populations in the Italian region (Aosta valley), indicating different evolutionary origins^28^. We selected a lowland and a highland population in each region (Swiss lowland: 46° 07′ 53″ N, 7° 03′ 51″ E, 500 m.a.s.l.; Swiss highland: 46° 01′ 36″ N, 7° 28′ 53″ E, 2000 m.a.s.l.; Italian lowland: 45° 42′ 12″ N, 7° 12′ 38″ E; 700 m.a.s.l.; Italian highland: 45° 49′ 21″ N; 7° 37′ 10″ E; 1950 m.a.s.l.).

Both lowland populations are associated with the crab spider *Thomisus onustus* (Fig. 1), whereas we did not observe these spiders in the highland plant communities. *T. onustus* has two reproductive periods, one in spring and another in autumn, and therefore depends on extended flowering seasons, making the alpine highlands an unsuitable habitat^63^. In both lowland populations, crab spiders hunt insects nearly exclusively on *B. laevigata* throughout its flowering season (during 3 years of field work only three *T. onustus* individuals were observed on other plant species). The spider abundance in the Swiss lowland population was measured three times (once in 2014 and twice in 2017) by checking around 100 *B. laevigata* plants for their association with a crab spider (94, 100 and 105 plants were checked at once).

*Biscutella laevigata* is self-incompatible^59^ and has a generalized pollination system. To characterize the pollinator guilds of the Swiss lowland and highland population, floral visitors were surveyed during the flowering time during 9 and 7 days, respectively. Observations of floral visitors were made by random walking across field sites for at least 1 h per day, whereas all observed visits were recorded to measure relative pollinator abundances. To identify pollinators, we captured some individuals and subsequently identified them at the genus or family level. Additionally we measured the pollination effectiveness by quantifying the pollen carryover and the number of visited flowers per visit. Pollination effectiveness was assessed for the orders Hymenoptera and Diptera, which included the most abundant visitors. To measure pollen carryover, we randomly chose ten plants per population and bagged inflorescences in bud stage. After flower opening, the bags were removed and all opened flowers were emasculated to avoid selfing. After single visits by floral visitors we counted the number of pollen grains on the stigmas of visited flowers. Pollen counts were done under a stereoscopic binocular microscope (Nikon Nature Scope, Nikon, Japan). Pollen carryover was measured for 13 flower visitors (seven individuals in the order Hymenoptera and six in the order Diptera) in the lowlands and for 25 visitors (five individuals in the order Hymenoptera and 20 in the order Diptera) in the highlands (some of the bagged plants were used to record more than one visit). Further, the number of visited flowers per single visit was recorded for 34 pollinators (20 individuals in the order Hymenoptera and 14 in the order Diptera) in the lowland population and 68 (18 individuals in the order Hymenoptera and 50 in the order Diptera) in the highland population.

To get an estimate of the degree of pollen limitation in the Swiss lowland population of *B. laevigata*, we measured the mean female fitness of the population and of some plants receiving supplemental hand-pollination. The mean fitness of the population was determined by measuring the relative seed set in 76 plants after the flowering period in 2014. Supplemental hand-pollination was done in an additional ten plants for three flowers per individual. As *B. laevigata* can develop two seeds per flower maximally^59^, the fitness was calculated as (number of seeds)/(2*number of flowers).

*B. laevigata* flowers are attacked by various different species of florivores, including *Plutella xylostella*, various species of Pieridae, and different species of Coleopterans. Florivore infestation rates were measured for the Swiss low- and highland populations in the years 2016 and 2014 respectively. We scanned 105 individuals in the lowlands and 56 individuals in the highlands for the presence of florivores on inflorescences. Although different beetle species occasionally feed on *B. laevigate* petals, we only recorded larvae (mainly caterpillars and beetle larvae) that feed on whole flowers and can drastically reduce plant fitness. Additionally, we measured plant fitness for all scanned individuals in the highland population after seed development. To control for differences in flower number between individuals, fitness was measured as relative seed set. As *B. laevigata* can develop two seeds per flower maximally^59^, the relative seed set was calculated as: (number of seeds)/(2*number of flowers). To test for an effect of florivory on plant fitness we conducted a *t*-test between infested and non-infested plants.

For all experiments, we used plants cultivated in a common garden environment from seeds collected from wild plants. *Biscutella laevigata* seeds were collected from 50 individuals in each of the four populations, except for the Italian highland population, where only 20 individuals with ripe fruits could be found. The seeds were germinated and experimental plants were grown outdoors in the Botanical Garden of Zurich. All plants were kept under netting before the start of flowering to avoid pollination and infestation with florivores until experimental use. For the ‘Local Adaptation’ experiment we used seeds from all four populations. The other experiments were conducted with plants cultivated from Swiss lowland seeds.

Experiments with pollinators and crab spiders were conducted on a meadow about 50 m from the Swiss lowland population. This allowed us to work with the natural pollinator guild and to collect crab spiders directly from *B. laevigata* flowers in the natural population. Only female spiders were used in the experiment, as they are the main foragers and are two to three times bigger than males^63,64^. All experiments were performed during the flowering season of *B. laevigata* in spring 2015 and 2016. For experimental florivore infestations, we used larvae of the diamondback moth *Plutella xylostella* (obtained from Syngenta, Stein AG, Switzerland), which is a common florivore species in Brassicaceae^65^.

### Interaction between crab spider and pollinator attraction

To determine which scent compound(s) attracted *T. onustus* to *B. laevigata* flowers, we conducted dual choice behavioral assays. Each of the three scent compounds was tested against an odorless control using a Y-tube olfactometer (15-cm arm length, 1-cm diameter, 45° angle; *n* = 30 *T. onustus* individuals per compound). Scent compounds were emitted from gray rubber septa (Supleco, Bellefonte, PA, USA) at emission rates similar to those of one *B. laevigata* individual (Supplementary Table 4). Septa were soaked for one hour in a solution of synthetic scent compounds in solvent and subsequently dried for about 2 h to reach constant emission rates. We used solutions of 7 μl ml^−1^ β-ocimene (mixture of isomeres, ≥90%, Sigma Aldrich, St. Louis, USA), 7 μl ml^−1^ 2-aminobenzaldehyde (≥98%, Sigma Aldrich, St. Louis, USA) and 2 μl ml^−1^ p-anisaldehyde (≥98%, Sigma Aldrich, St. Louis, USA). Odorless control septa were soaked in pure solvent. Scented and control septa were placed at the two ends of the Y-tube. Each end was connected to a membrane pump (Personal Air Sampler, SKC, USA), which pushed air into the tube at a rate of 150 ml min^−1^. After each choice by a spider the Y-tube was cleaned with acetone and water and the positions of the two septa were exchanged. All behavioral assays were analyzed using binomial tests. The proportion of choices for one category was tested against an expected value of 0.5.

As crab spiders showed a strong preference for β-ocimene in the dual-choice behavioral assays, we then tested for an association between the emission of this compound and the presence of crab spiders in the natural *B. laevigata* population (Swiss lowland). We collected scent from the inflorescences of 94 randomly selected individuals (volatile sampling and analysis as described below), which were in full flower. For each of these individuals we additionally recorded if there was a female *T. onustus* on the inflorescence or not (33 individuals with spider and 69 without). To test for an association between spider distribution and β-ocimene emission we used a binomial logistic regression. The presence of spiders (absent vs. present) was fitted as the response and the amount of β-ocimene as explanatory variable.

To test for a trade-off between pollinator attraction and predator avoidance in *B. laevigata*, we measured whether β-ocimene was also attractive to bees. To do so we conducted a dual-choice behavioral assay in the field, presenting bees with a choice between a plant with augmented β-ocimene emission and a plant with unmanipulated emission (control). The two plants were placed 20 cm apart on the meadow and each landing of a bee (all flower visitors within Apidae) on an inflorescence was recorded. In total we observed 35 landings; after each landing the position of plants was exchanged. Scent was augmented by fixing a rubber septa emitting β-ocimene on the inflorescence, control plants received odorless septa (septa preparation as described above). Scented and odorless septa were exchanged between plants after 17 landings. Data were analyzed using a binomial test. The proportion of choices for one category was tested against an expected value of 0.5.

We used field plot experiments to investigate the influence of crab spiders on pollinator behavior and its association with floral traits. Six plots of 36 *B. laevigata* plants (6 × 6 plants, 40 cm distance between plants) were placed in the open field for five sunny days under one of two treatments: (a) no crab spiders in the plot; (b) 9 to 12 crab spiders hunting in the plot. For each treatment, three replicates were conducted leading to a sample size of 108 plants per treatment. For all plants used in this experiment, we measured floral scent and flower size (measurement of floral traits described below) 1 day before the plots were placed at the field site. These measurements were conducted in the greenhouse under standardized light and temperature conditions. Additionally, to count flower number, we marked the lowest flower on the first day and then counted the pedicles from markings to buds at the last day of the experiment. Pollinators were observed during 17.5 h in total (several hours per day); plots were observed continuously in rotation with each plot observed for about 5 min before moving to the next plot. We only noted visits by bees (all flower visitors within Apidae), as they were the dominant pollinators in our field site with respect to abundance and pollinator effectiveness (Supplementary Tables 1, 2). A visit was defined as a landing on an inflorescence of one *B. laevigata* individual. In treatment (b), spiders were allowed to move freely inside the plots. Spider abundance was monitored regularly and whenever the number of spiders hunting on flowers dropped below 9, a new individual was released (spiders occasionally hid somewhere on the ground but never moved to another plot). The position of each crab spider inside the plots was noted three times a day. For each *B. laevigata* individual, we quantified the attendance of crab spiders as the sum of time intervals in which a spider was observed on the inflorescence. For statistical analysis, floral traits were standardized by *z*-transformation within plots, which allows comparison of regression coefficients between traits^66^. To test for differences of bee attraction between treatments, we used a generalized mixed model with Poisson distribution. The number of visits by bees was included as the response, floral traits and treatment as explanatory variables, and plot as a random effect. Subsequently, we used analysis of variance for the model objects produced by a generalized mixed model to test for significant treatment × trait interactions, which indicate differences in the regression coefficients between treatments. To calculate regression coefficients for the floral traits for which a significant trait × treatment interaction was found, we fitted a generalized mixed model with Poisson distribution for each treatment separately. To measure regression coefficients for the traits that did not show a treatment × trait interaction, we fitted a model with the data from both treatments. In these models, the number of visits by bees was included as the response, floral traits as explanatory variables and plot as a random effect. Further, to test for the direct effect of crab spiders on bee attraction, we included the spider attendance per plant as an explanatory variable in the model (for treatment (b) only).

### Tritrophic interaction

We tested for antagonistic and mutualistic effects of crab spiders on plant fitness in the absence and presence of florivores. Twenty-five plots of four plants each (20 cm distance between plants) were placed in the open field for 2–4 days (depending on the weather). Within each plot, individuals were randomly assigned to one of the following treatments: (a) control; (b) a crab spider on inflorescence; (c) infestation with florivores; (d) a crab spider on inflorescence and infestation with florivores. Spiders were placed on the inflorescences for treatments (b) and (d) at the beginning of the experiment. Every morning we further placed three *P. xylostella* larvae (larval stages L2 to L3) on the plants in treatments (c) and (d) and counted the remaining number of larvae in the evening. In these treatments, we additionally counted the number of damaged flowers and buds on the last experimental day to quantify total floral damage by florivores. We checked the position of spiders in the plots three times a day, and we returned them to the right plants if necessary (in five occasions spiders had changed from a plant without florivores to a neighboring plant with florivores). Additionally, we noted which spiders were feeding on a prey, and if so the type of prey (pollinator or *P. xylostella* larvae). About four weeks after the experiment, we counted the number of developed seeds as an estimate of the female component of fitness. We did not assess flower visitation by pollinators as an estimate of male fitness, as in this specific case it would not account for pollen reduction by florivores. We used a linear mixed model to analyze the effect of crab spiders and florivory on female fitness. Spider presence (present vs. absent) and florivore infestation (present vs. absent) were fitted as explanatory variables. In addition, we included flower number as a covariate to control for differences in flower number between plants. Plot was included as a random effect. Linear mixed models were also used to analyze the effect of spider presence on the number of florivores on the inflorescence (mean number per plant individual from day 1 to 5) and floral damage. The spider presence (present vs. absent) was fitted as explanatory variable and plot as random effect in both analyses.

Because spiders had a positive effect on plant fitness in florivore-infested plants, we quantified the proportion of florivores in the diet of crab spiders in the natural *B. laevigata* population. We searched all plants in the population for crab spiders and identified all their prey as florivores (caterpillars) or pollinators (bees or syrphid flies) during 5 days distributed over the whole flowering season (50–300 flowering plants at the time). In total, we found 17 spiders with a prey item. Furthermore, to test if crab spiders prefer hunting on florivore-infested plants, we scanned 105 plants for the presence of florivores and crab spiders on inflorescences. To analyze this data we used a binomial logistic regression with the presence of spiders (absent vs. present) as response and the presence of florivores (absent vs. present) as explanatory variable.

To test for the inducibility of the spider attractant β-ocimene by florivory, we grew 68 plants from 34 half-sib families (two plants per family, seeds from Swiss lowland population). Individuals from the same family were assigned randomly to one of two treatments: (a) control; (b) infestation with *P. xylostella* larvae. We quantified constitutive volatile emission in all plants before infestation (volatile sampling and analysis as described below). The next day, plants in treatment (b) were infested with larvae in stage L2 and about 24 h afterwards the scent collection was repeated. Measurements were made over three consecutive weeks, and we sampled the same number of plants per treatment each week. In total, we measured 28 plants per treatment (from 6 families we could only measure one individual). Scent collection was conducted in the greenhouse under standardized light and temperature conditions. To measure inducibility of β-ocimene, the change in scent emission from the first to the second scent collection was calculated in ng l^−1^ flower^−1^. This variable was the response in a linear mixed model with treatment (infested vs. control) as explanatory variable and the half-sib family as random effect. For graphical display we additionally calculated the inducibility as percentage change in scent emission as this measure contains more information about the biological relevance of inducibility.

To test whether crab spiders showed a preference for florivore-infested plants, we used a dual-choice behavioral assay. We presented 29 crab spiders with pairs of plants with similar flower number within a pair (maximal difference of eight flowers out of 50 to 112 flowers, 15 pairs in total). One plant per pair was selected randomly and infested with five* P. xylostella* larvae (stage L2) 2 days before the behavioral assay, while the other plant (control) was left uninfested. Plants were presented to crab spiders at a distance of 20 cm. The positions of the infested and the control plant were switched after each trial to control for wind direction and light conditions. Crab spiders were placed on a wooden stick in the middle of the two plants. The top of the stick was at flower level (at the middle when plants had different inflorescence heights), allowing crab spiders to directly move to inflorescences. The first plant they moved to and settled on flowers was noted as their choice. Data were analyzed using a binomial test. The proportion of choices for one category was tested against an expected value of 0.5.

### Local adaptation to crab spiders

To test for differences in the inducibility of β-ocimene between low- and highland *B. laevigata* populations, we quantified volatile emission in plants before and after infestation with *P. xylostella* larvae. We sampled 30 plants per population from the Swiss lineage and 25 from the Italian lineage. On 1st day, we collected scent from the same number of plants from the lowland and highland populations per region (volatile sampling and analysis as described below). The next day, all plants were infested with five *P. xylostella* larvae (in stage L2) and about 24 h after infestation the volatile sampling was repeated. Scent collection was conducted in the greenhouse under standardized light and temperature conditions. Inducibility of β-ocimene was calculated as difference in the emitted amount per flower before and after florivore infestation. We fitted a linear model with the altitude (lowland vs. highland) and the region (Swiss vs. Italian) as explanatory variables to test for differences in inducibility due to differences in spider occurrence and region, respectively. Additionally, the absolute emission values before and after infestation were analyzed with the same model.

Because we found a positive fitness effect of crab spiders on infested plants, we investigated local adaptation to crab spiders by testing for an increased attractiveness of lowland and highland plants after florivore infestation. We used dual-choice behavioral assays; each spider was presented with one lowland and one highland plant from the same region (Italy or Switzerland). Two assays were conducted; one with infested plants and one with control plants. Plants were infested with five *P. xylostella* larvae (stage L2) 2 days before the behavioral assay. During the assays, lowland and highland plants were presented to crab spiders at a distance of 20 cm, and the positions of the two plants were switched after each trial to control for wind direction and light conditions. Crab spiders were placed on a wooden stick in the middle of the two plants. The top of the stick was at flower level (at the middle when plants had different inflorescence heights) allowing crab spiders to move directly to inflorescences. The first plant they moved to and settled on flowers was noted as their choice. For each comparison (Italian and Swiss region, infested and control), we tested 19–24 pairs of plants. Behavioral assays were analyzed using binomial tests. The proportion of choices for one category was tested against an expected value of 0.5.

### Measurement of floral traits

For scent collection in the field, we used the dynamic headspace collection method^67^. Inflorescences were inserted into polyethylene terephtalate cooking bags which were closed around the stem with a wire. To trap volatiles, we used glass tubes filled with ca. 20 mg of Tenax TA (Tenax TA 60/80, Supelco, Bellefonte, PA, USA), that we inserted into the bag from the other side and attached to a Micro Air Sampler (PAS-500 Micro Air Sampler, Spectrex, Redwood City, CA, USA) using a silicon tube. Subsequently, air was pulled through the tubes filled with Tenax at a flow rate of 150 ml min^−1^. We collected scent samples between 11^00^ and 15^00 ^hrs. Afterwards, we stored the tubes in sealable glass tubes (7 ml, Sigma-Aldrich, Buchs, Switzerland).

For scent collection from inflorescences in the greenhouse we used the push-pull headspace scent collection method^68^. We enclosed inflorescences in glass cylinders (5 cm diameter, 25 cm height) that were previously treated with sigmacoate (Sigma-Aldrich, Buchs, Switzerland). We closed the bottom of the cylinder with a teflon plate with a central hole that allowed for the insertion of the stalk without injuring it. For volatile collection we inserted glass tubes filled with Tenax (as described above) into a opening in the glass cylinder and connected vacuum pump (DC06/04/20 F, Fürgut GmbH, D-88459 Tannheim) using a silicone tube. The pump pulled air through the Tenax tubes at a flow rate of 150 ml min^−1^. Simultaneously, air was pushed with the same flow rate into the glass cylinder through a second opening. A second Tenax tube (filled with Tenax GR 60/80, Scientific Instrument Services, Old York, NJ, USA) was used to clean the incoming air. To be able to calculate volatile amounts per flower, we counted the number of flowers inside the cylinder during scent collection. We collected scent between 11^00^ and 15^00 ^hrs. Until chemical analysis with gas chromatography, Tenax tubes were stored at −30 °C in a freezer.

We used gas chromatography with mass selective detection for chemical analysis of scent samples. Samples were injected into a GC (Agilent 6890 N; Agilent Technologies, Santa Clara, CA, USA) with a Gerstel thermodesorption system (TDS3; Gerstel, Mühlheim, Germany), employed with a cold injection system (KAS4; Gerstel). The GC was equipped with a DB-5 column (0.32 mm ID, 0.25 lm film thickness, 30 m length). Helium was used as carrier gas at a flow rate of 2 ml min^−1^. We identified volatiles by comparing of MS spectra obtained from the samples with those of synthetic standard compounds, analyzed previously. We used the same standard compounds for compound quantification through dose-response curves obtained for each floral volatile using characteristic ions^68^. Because the floral scent of *B. laevigata* is strongly dominated by the monoterpene β-ocimene (Z-isomere and E-isomere, ratio about 1:3) and the two aromatics p-anisaldehyde and 2-aminobenzaldehyde we only considered these three compounds for analysis and behavioral assays (Supplementary Table 5). As the two isomers of β-ocimene were strongly correlated to each other (Pearson correlation analysis, *r* = 0.97, *P* < 0.001) we used their sum for statistical analysis. For the same reason the sum of the two aromatic compounds p-anisaldehyde and 2-aminobenzaldehyde was used (Pearson correlation analysis, *r* = 0.61, *P* < 0.001).

We measured petal length and width in three fully opened flowers per individual (one petal per flower). Subsequently, we used means of petal length and width to estimate the corolla size per flower as: π*length*width (formula for an ellipse multiplied by 4 for the four petals).

### Data availability

The data are available from the ZORA server of the University of Zürich under 'Supplemental Material' at the following link: 10.5167/uzh-150220.

## Electronic supplementary material

Supplementary Information(PDF 270 kb)
