# Supplementary material for: Crab spiders impact floral-signal evolution indirectly through removal of florivores
Source: Nat Commun. 2018 Apr 10;9:1367. doi: 10.1038/s41467-018-03792-x (PMC5893632; doi:10.1038/s41467-018-03792-x)
Supplement: Supplementary file 1 — Supplementary Information(PDF 270 kb) [file 41467_2018_3792_MOESM1_ESM.pdf]

Supplementary Information

Crab spiders impact floral-signal evolution through removal of florivores

Knauer et al.

Nature Communications

Supplementary Table 1: Relative abundance of pollinator guilds for the Swiss lowland and highland populations of *B. laevigata*. Relative abundances for whole insect orders are given in bold.

Insect taxa		Relative abundance	
Order	Family	Lowland	Highland
Hymenoptera	Apidae	0.406	0.016
	Xyelidae	0.007	-
	Megalodontidae	0.004	-
	Tenthredinidae	0.029	0.271
	Formicidae	-	0.062
	Total	**0.446**	**0.348**
Diptera	Syrphidae	0.219	0.135
	Platypezidae	-	0.163
	Scatophagidae	-	0.186
	Total	**0.219**	**0.485**
Lepidoptera	Lycaenidae	0.087	0.007
	Nymphalidae	0.047	0.005
	Pieridae	0.004	0.009
	Torticidae	0.029	0.019
	Noctuidae	0.004	-
	Total	**0.171**	**0.040**
Coleoptera	Cerambycidae	0.007	0.107
	Scarabaeidae	0.004	-
	Cantharidae	0.004	-
	Elateridae	-	0.005
	Total	**0.015**	**0.112**
Hemiptera	Pentatomidae	**0.131**	**0.014**

Supplementary Table 2: Mean (± s.e.) pollen carryover and number of visited flowers for insect visitors from the order Hymenoptera and Diptera in the Swiss lowland and highland populations.

Population	Insect taxa	Pollen carryover	Number of visited flowers
Lowland	Hymenoptera	10.6 ± 1.4	8.3 ± 1.4
	Diptera	6.9 ± 2.0	4.9 ± 0.7
Highland	Hymenoptera	14.0 ± 1.3	3.7 ± 0.7
	Diptera	5.4 ± 0.5	5.1 ± 0.4

Supplementary Table 3: Mean (± s.e.) amounts [ng l^{-1} $flower^{-1}$] of the three main compounds of *B. laevigata* before infestation with florivores (constitutive emission) and after infestation. Additionally the induced amount was calculated as the change in emission through florivory (N = 31 for Swiss lowland population, N = 30 for Swiss highland population, N = 24 for Italian lowland population, N = 26 for Italian highland population).

Compound	Constitutive			
	Swiss		Italian	
	Lowland	Highland	Lowland	Highland
β-Ocimene	3.8 ± 0.4	3.1 ± 0.3	3.7 ± 0.4	3.0 ± 0.3
2-Aminobenzaldehyde	9.9 ± 0.7	3.7 ± 0.5	8.7 ± 1.4	4.1 ± 0.8
p-Anisaldehyde	5.4 ± 0.4	0.7 ± 0.3	0.2 ± 1.4	0.8 ± 0.2
	Infested			
	Swiss		Italian	
	Lowland	Highland	Lowland	Highland
β-Ocimene	5.9 ± 0.4	4.2 ± 0.4	5.7 ± 0.7	3.9 ± 0.4
2-Aminobenzaldehyde	9.9 ± 1.0	1.9 ± 0.4	4.0 ± 0.2	1.4 ± 0.4
p-Anisaldehyde	4.1 ± 0.5	0.4 ± 0.07	0.7 ± 0.03	0.3 ± 0.06
	Induced (infested - constitutive)			
	Swiss		Italian	
	Lowland	Highland	Lowland	Highland
β-Ocimene	2.1 ± 0.3	1.2 ± 0.2	2.0 ± 0.5	0.9 ± 0.2
2-Aminobenzaldehyde	0.005 ± 0.9	-1.9 ± 0.3	-4.5 ± 1.2	-2.8 ± 0.5
p-Anisaldehyde	-1.3 ± 0.3	-0.4 ± 0.09	-0.03 ± 0.04	-0.5 ± 0.1

Supplementary Table 4: Amounts (ng l^{-1} $inflorescence^{-1}$) of the three main volatile compounds of *B. laevigata* emitted by plants and septa. Scent was collected from 95 plants in the Swiss lowland population and 5 septa as described in the main manuscript.

Compound	Plant		Septa
	mean ± s.e.	maximum	mean ± s.e.
β-Ocimene	20.0 ± 2.0	91.0	39.8 ± 1.6
2-Aminobenzaldehyde	24.8 ± 2.6	106.7	46.5 ± 1.0
p-Anisaldehyde	26.4 ± 2.4	153.4	39.6 ± 0.7

Supplementary Table 5: Mean (± s.e.) amounts [ng l^{-1} $flower^{-1}$] of all floral scent compounds identified in *B. laevigata* from the Swiss lowland population (N = 31).

Compound	Amount
**Aromatic compunds**	
Benzaldehyde	0.60 ± 0.06
2-Aminobenzaldehyde	9.9 ± 0.7
p-Anisaldehyde	5.4 ± 0.4
**Terpenoids**	
Eucalyptol	0.20 ± 0.02
(*Z*)-β-Ocimene	1.0 ± 0.1
(*E*)-β-Ocimene	2.8 ± 0.3
